# Shaker potassium channel mediates an age-sensitive neurocardiac axis regulating sleep and cardiac function in *Drosophila*

**DOI:** 10.1007/s10522-026-10386-4

**Published:** 2026-01-18

**Authors:** Kishore Madamanchi, Dalton Bannister, Ariel Docuyanan, Shruti Bhide, Girish C. Melkani

**Affiliations:** 1 Department of Pathology, Division of Molecular and Cellular Pathology, Heersink School of Medicine, The University of Alabama, Birmingham, AL 35294 USA; 2 Department of Biology, Molecular Biology Institute, San Diego State University, San Diego, CA 92182 USA; 3 UAB Nathan Shock Center, Birmingham, AL 35294 USA

**Keywords:** Age-linked cardiac dysrhythmia, Sleep-circadian cycle, Voltage-gated potassium channels, *Drosophila Shaker* mutation, Neurocardiac axis, Sleep-cardiac linkage, Time-restricted feeding

## Abstract

The *Shaker* (*Sh*) gene in *Drosophila melanogaster* encodes a voltage-gated potassium channel that regulates neuronal excitability and is well known for its role in sleep regulation; however, its contribution to cardiac physiology and neurocardiac communication remains insufficiently explored. In this study, we investigated how two *Sh*-mutations (*Sh*^*mns*^ and *Sh*^*5*^) influence heart function and sleep/circadian behaviors to identify potential age-dependent neurocardiac interactions. Cardiac performance and sleep/activity were examined across aging cohorts under normal conditions, circadian disruption, and with or without time-restricted feeding (TRF). *Sh*^*mns*^ mutants exhibited progressive cardiac decline with age, including increased heart period, elevated arrhythmicity, extended systolic and diastolic intervals, reduced contraction rate, and overall impaired cardiac output, along with disorganization of actin-rich myofibrils. These defects paralleled severe sleep loss and hyperactivity, suggesting a tight link between sleep/circadian dysregulation and cardiac impairment. Circadian misalignment further worsened both behavioral and cardiac deficits, whereas TRF partially improved select abnormalities, indicating feeding-time modulation of *Sh*-related phenotypes. Tissue-specific knockdown of *Sh* in cardiac and neuronal tissues recapitulated key mutant features, and notably, neuronal knockdown alone impaired cardiac behavior, supporting a functional neurocardiac regulatory axis mediated by Shaker-dependent electrical signaling. Together, these results demonstrate that Shaker channels contribute to an age-sensitive interplay between sleep/circadian regulation and cardiac homeostasis in *Drosophila*. Although direct extrapolation is limited, parallels with KCNA1-associated cardiac and neuronal abnormalities in humans suggest conserved Kv channel functions in neurocardiac dysfunction. Overall, this study identifies Shaker as a critical mediator of aging-, feeding-, and circadian-sensitive cross-tissue regulation of cardiac function, providing broader insight into mechanisms underlying neurocardiac communication. Our study establishes Shaker as a critical mediator of aging, circadian-sensitive, cross-tissue physiological regulation of cardiac function.

**Graphical abstract:**

Highlights the role of the *Shaker* (*Sh)* potassium channel in linking neuronal and cardiac function in *Drosophila*. *Sh*^*mns*^ (Mini sleep) mutations cause age-dependent cardiac dysfunction and severe sleep loss (doted black head arrow). Circadian disruption worsens these effects, while time-restricted feeding (TRF) partially rescuing them (doted green blunt head arrow). Neuronal-specific *Sh* knockdown impairs heart function, revealing a neurocardiac axis critical for sleep and cardiac homeostasis.

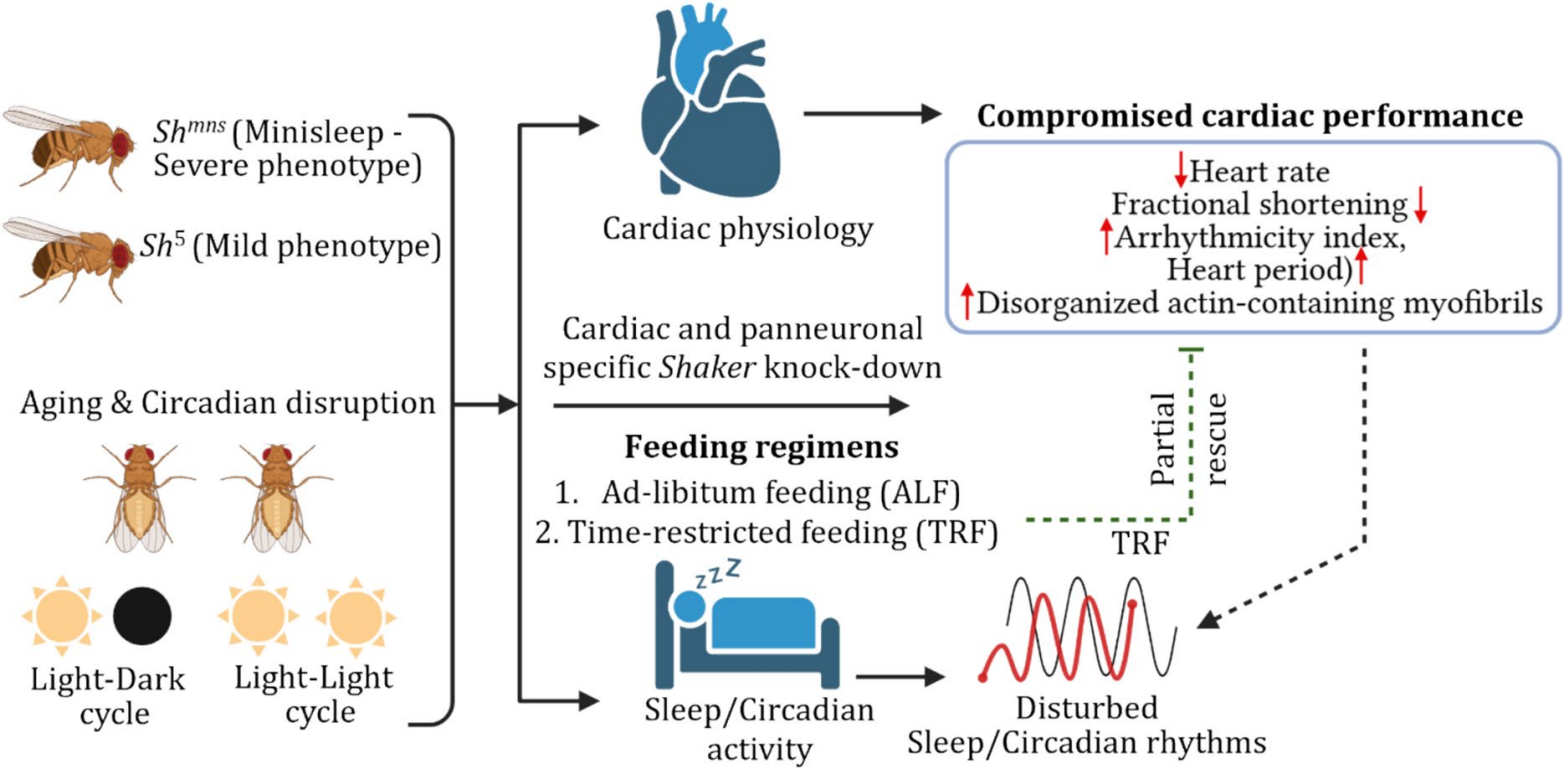

## Introduction

Voltage-gated potassium channels (Kv) are vital regulators of electrical excitability across animal species. Kv1/KCNA1 (potassium voltage-gated channel subfamily A member 1) channels in mammals expressed in both neuronal and cardiac tissues, emphasizing the evolutionary conservation across systems (Glasscock 2019). Dysfunction of these ion channels leads to neuronal hyperexcitability, cardiac abnormalities, and autonomic dysregulation as seen in KCNA-inked episodic ataxia type 1 (EA1) (D’Adamo et al. 2015), and seizure influenced neurocardiac instability (Paulhus et al. 2020). In *Drosophila melanogaster* (referred as *Drosophila*)*,* the *Shaker* (*Sh*) gene, orthologs of Kv1/KCNA1 initially identified by its characteristic leg shaking phenotype when kept under ether anesthesia (Kaplan and Trout 1969; Kim and Nimigean 2016). This gene encodes the α-subunit of the voltage-gated potassium channel (Kv), essential for regulating neuronal excitability by mediating membrane repolarization after action potentials (Papazian et al. 1987; Tempel et al. 1987) through rapidly inactivating A-type Kv channel (Pongs et al. 1988). *Shaker* channels are primarily expressed in the axons and synaptic terminals of the *Drosophila* nerves (Cirelli et al. 2005), where they initiate action potentials, modify synaptic transmission and influence neuronal firing patterns (Kim et al. 2020).

Several *Shaker* mutants, including mini sleep (*mns*) and *Shaker*-*5* (*Sh*^*5*^) in *Drosophila,* exhibit significant reduction in sleep duration (Cirelli et al. 2005; Bringmann 2019), and abnormal neuronal function (Bushey et al. 2007). These *Shaker* mutants extensively used to study the metabolic stress, sleep regulation and aging, despite significant work on their circadian and neuronal roles, the impact of Shaker channel dysfunction on cardiac physiology is poorly understood. The significance of *Shaker* gene location on X-chromosome exacerbating stronger phenotypes in case of males (hemizygous) further adding additional layer of biological variability, which is not sufficiently explored in relation to cardiac function and sleep-cardiac interaction. Though studies using mammalian model system on Kv1.1 provide essential conceptual similarities and differences between insect’s and vertebrate’s cardiac organization and autonomic regulation we believe it is essential to characterize the Shaker dependent cardiac function in *Drosophila* model system.

Since *Drosophila* heart is myogenic tube with well-defined neuronal connections, and conserved Kv channel biology this model system provides a valuable system to explore neurocardiac interaction (Dulcis and Levine 2003, 2005). Previous studies reported that circadian disruption, feeding cues and altered neuronal firing can influence the cardiac function in flies (Gill et al. 2015), but the specific role of Shaker channel involvement in cross tissue interaction is still unknown. Previously no one has examined whether *Shaker* sleep mutants also exhibit cardiac abnormalities, nor how environmental factors like feeding rhythms and light cycles interact with *Shaker* mutations to influence cardiac performance.

In the current study, we aim to investigate whether loss of Shaker ion channel function disrupts cardiac physiology, Sleep/activity(wake) behavior and in age dependent manner. We compared two different *Shaker* mutant alleles (*Sh*^*mns*^ and *Sh*^*5*^) and evaluated their genetic interactions using trans-heterozygotes. Further we employed tissue specific RNAi procedure to determine whether *Shaker* directly influences the heart function by impacting cardiomyocytes (cell autonomous) or indirectly through neuronal circuit (non-cell autonomous) conditions. Further, we tested how disruptions in circadian rhythms (constant light-24 h) and feeding timings (time restricted feeding-TRF) influence behavior and cardiac outcomes in *Shaker* mutants. We believe these approaches can address whether a single Kv channel can coordinate circadian, neuronal and cardiac network and establish *Drosophila* as an ideal model system for exploring evolutionary conserved neurocardiac channelopathies.

## Materials and methods

### Drosophila stocks, rearing conditions, circadian disruptions and feeding-fasting regimens

Flies were reared on a standard laboratory diet consisting of 11 g/L agar, 30 g/L active dry yeast, 55 g/L yellow cornmeal, 72 mL/L molasses, 8 mL/L 10% nipagin, and 6 mL/L propionic acid. All the flies (*Shaker*^*mns*^, BDSC:53347 and BDSC:31680; *Shaker*^*5*^, VDRC:104474; controls w^1118^, BDSC:5905; attP2, BDSC:36303; attP40, BDSC:36304; driver lines *Elav-Gal4*, BDSC:458 and *Hand-Gal4*) were maintained at a controlled temperature of 23 °C with 50% relative humidity under a 12-h light/12-h dark (LD) cycle (Pasam et al. 2025). Newly eclosed adults were collected and sex-separated into groups of 25–30 individuals on the third day post-eclosion. Feeding and light exposure regimens commenced on the seventh day. In the LD condition, flies exposed to a 12-h light/12-h dark cycle, whereas those in the light/light (LL) group were exposed to continuous light (24 h light) (Reinhard et al. 2024). Flies designated for ad libitum feeding (ALF) or time-restricted feeding (TRF) (Lundell et al. 2020; Livelo et al. 2023). were provided access to standard diet vials beginning at zeitgeber time zero (ZT0), which corresponded to 8:00 AM (light onset). At 8:00 PM (light offset), ALF flies remained on their regular diet, while TRF flies were transferred to vials containing 1.1% agar. All experimental flies were transferred to fresh media every three days (Villanueva et al. 2019). To investigate the role of *Shaker*^*mns*^ and *Shaker*^*5*^ alleles on sleep/circadian activity and cardiac function, we have implemented the LD and LL, ALF and TRF interventions (Table 1), to evaluate their physiological impact on wild-type and mutant flies.

**Table 1:** Represent the food and light entrainments applied to understand their impact on *Shaker* gene knockdown

Environmental Cues and timings			Food	
			Day	Night
Light	Light-8 AM–8 PM Dark-8 PM–8 AM	LD-12/12 h	Standard diet	Standard diet
		LL-24 h	Standard diet	Standard diet
Food	Light-8 AM–8 PM	ALF-24 h	Standard diet	Standard diet
	Dark-8 PM–8 AM	TRF-8 PM–8 AM	Standard diet	1.1% agar

For the cardiac and neuronal specific function of shaker gene the fly stocks were ordered from the Bloomington *Drosophila* Stock Center (BDSC) and the Vienna *Drosophila* Resource Center (VDRC). The stocks include UAS-RNAi lines for the *Shaker* gene (BDSC: 53347; BDSC: 31680 and VDRC:104474), controls (attP2, BDSC: 36303 and attP40, BDSC: 36304). Cadiac-specific driver *Hand-Gal4* was obtained from Dr. Olson’s laboratory, (University of Texas Southwestern Medical Center, Dallas, TX, USA) and another one for panneuronal expression (*Elav-Gal4*; BL#458). The RNAi system uses small interfering RNA (siRNA) strands that are complementary to a gene of interest to experimentally silence its expression. The *Shaker* knockdown was accomplished through UAS-RNAi lines targeting Shaker transcripts BL#53347(attP40 insertion) and BL#31680 (attP2 insertion). The siRNA fragments bind to the mRNA of *Shaker* gene and degrade them, thereby preventing mRNA translation (Ichim et al. 2004). We used BDSC: 36303 control line carrying an empty attP2 vector inserted in the 3rd chromosome, while the BDSC: 36304 control line carrying an empty attP40 vector in the 2nd chromosome, which ensures the phenotypes observed in the experiments appeared from *Shaker* gene silencing and not from insertional effects at specific sites. The flies from each UAS-RNAi *Shaker* and control line were crossed with virgin female flies from the *Hand-Gal4* and *Elav-Gal4* for cardiac and panneuronal driver lines respectively. The F1 progeny flies were collected (Villanueva et al. 2019; Guo et al. 2024). Male and female progeny flies were separated and maintained in a standard food at 25 °C and transferred onto fresh food every 3–4 days throughout the study. Using three-week-old (mid age) and five-week-old (early aging) male and female flies we have performed all the experiments with indicated number of flies in each experiment.

### Cardiac physiology

Progeny from each line was collected at three-weeks or five-weeks of age for heart physiology analysis. Semi-intact heart dissections were performed to make the heart visible for recording (Fink et al. 2009; Guo et al. 2024). Flies were made unconscious with CO_2_ gas and fixed on their backs to petri dishes with petroleum jelly. Under a light microscope, the head and thorax were removed, followed by a small incision at the apex of the abdomen. Plates were then flooded with warmed, aerated artificial hemolymph (Trehalose 5 mM, supplemented with physiological saline containing 108 mM NaCl, 5 mM KCl, 2 mM CaCl_2_, 8 mM MgCl2, 1 mM NaH_2_PO_4_, 4 mM NaHCO_3_, 15 mM HEPES and sucrose 10 mM with pH 7.2) to ensure hearts maintained myogenic activity during recording. which is continuously aerated to maintain myogenic contraction. The top of the abdomen was then removed, followed by the intestine and fat, exposing the heart. After that recordings were taken using an immersion microscope lens with an attached high-speed camera. Heart physiology was recorded between the second and third body segments to ensure consistency across flies. Recordings were made using a Promon u750 microscope camera in B&W at 200 frames/second, for 30 seconds (Fink et al. 2009; Gill et al. 2015; Guo et al. 2024). Videos were analyzed using semi-automated heart analysis (SOHA) software and data output was organized in excel files with M-mode records (Cammarato et al. 2015; Gill et al. 2015). The SOHA data we analyzed included heart period, arrhythmicity index, systolic and diastolic intervals, systolic and diastolic diameters and fractional shortening (Guo et al. 2024). The sample size for heart analysis is 15–20 per group. The SOHA analysis performed blind conditions. Using Prism 10 software, the SOHA output variables for experimental lines were statistically compared to the control lines using one-way analysis of variance (ANOVA). Significance was set at *p* < 0.05, and Tukey’s multiple comparisons test was used for each data set.

### Cytological analysis

As previously described, the fly bodies were maintained for 20 min in a 4% paraformaldehyde (PFA) solution after the heads, legs, and wings were removed for the cytological test (Guo et al. 2024; Abou Daya et al. 2025). The fixed samples were then incubated for 15 min between each of the three PBS washes. The thoraces were longitudinally oriented in a cryomold using OCT (Fisher Scientific #4585) and flash-frozen on dry ice. Following cryosectioning to reach a thickness of 30 µm, three further washes in 1 × PBS were carried out, each requiring a 15-min incubation period. Samples were rinsed three times in PBS to assess structural abnormalities following a 30-min staining process with 0.1-µm Alexa-594-Phalloidin to detect actin-containing myofibrils. The quantification of myofibril stained with phalloidin was performed using Image J using images from multiple sections for each group (Guo et al. 2024). In disorganized hearts the F-actin -containing myofibrils found to have more gaps than organized hearts in controls. Sample size n = 5 flies per genotype.

### Sleep-circadian activity

As previously used, individual flies were anesthetized with CO_2_ and placed in glass tubes with food at one end and cotton plug at another end for aeration. These tubes were placed in the *Drosophila* activity monitor system (DAMS, Trikinetics) (Abou Daya et al. 2025). These monitors were then placed in incubators to regulate temperature and humidity (25 °C and 50% relative humidity). The data analysis was performed using Clock lab software, DAM analysis, outliers were removed with modified Z-score method in Microsoft Excel, and final graphs were plotted using Graph Pad Prism l0 software. We measured sleep, activity during daytime, nighttime and overall (24 h). Raw data was used to analyze sleep (immobility of a fly > 5 min), activity (fly crossing of an infrared beam at the center of DAM), Average sleep/activity behavior of individual fly from each genotype and condition in 24 h (ZT0-ZT24) was calculated over 5 days (Yadav et al. 2025; Abou Daya et al. 2025). Data from the day the individual flies were introduced to the DAMS monitors was not included in the average to allow for adaptation. Sample size n = 25–30 flies per genotype and age group. One-Way ANOVA is used for statistical analysis.

## Statistical analysis

**Fig. 1:**
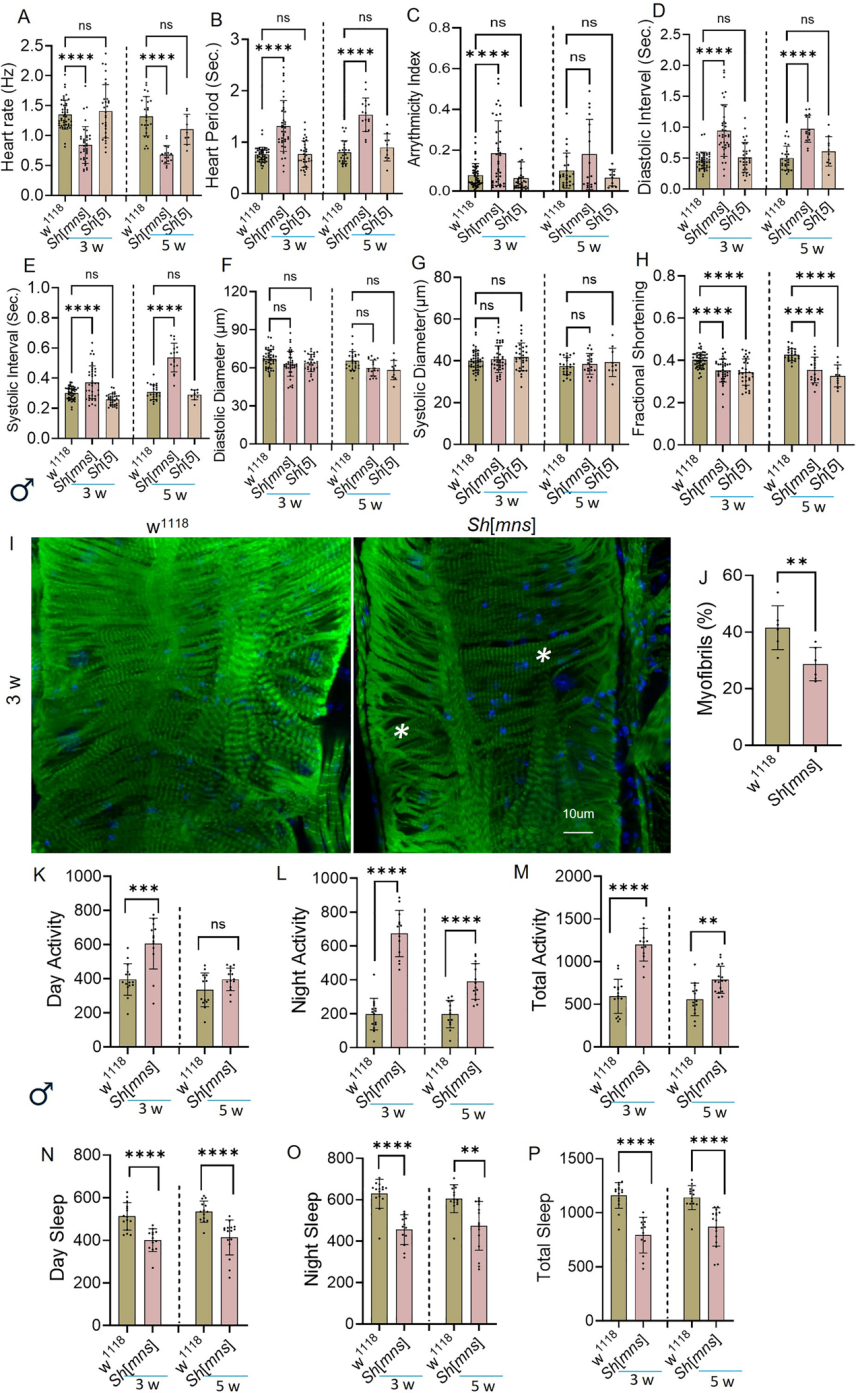
*Sh*^*mns*^ and *Sh*^5^ mutant alleles inversely impact heart function. The cardiac physiology and sleep/activity of three-week and five-week-old male flies were analyzed for the *Shaker* mutants mini-sleep (*Sh*^*mns*^) and 5 (*Sh*^5^) and compared to that of the age and sex-matched control line w^1118^. Different variables of heart function were measured; with **A**–**H** represent data collected from three-week and five-week-old male *Sh*^*mns*^ w^1118^ and *Sh*^5^ flies. Phalloidin staining to show myofibril percentage in heart muscle, measured using Image J. **K**–**P** sleep/activity analysis w^1118^ and *Sh*^*mns*^ at three-weeks and five-weeks of age. Statistics: Heart physiology and sleep/activity analysis performed by One-Way ANOVA with Šidák multiple comparison test. Myofibril percent was calculated based on Image J using an Unpaired t-test. *p* > 0.05 (ns), *p* < 0.02 (*), *p* < 0.0002 (***), *p* < 0.0001(****)

**Fig. 2:**
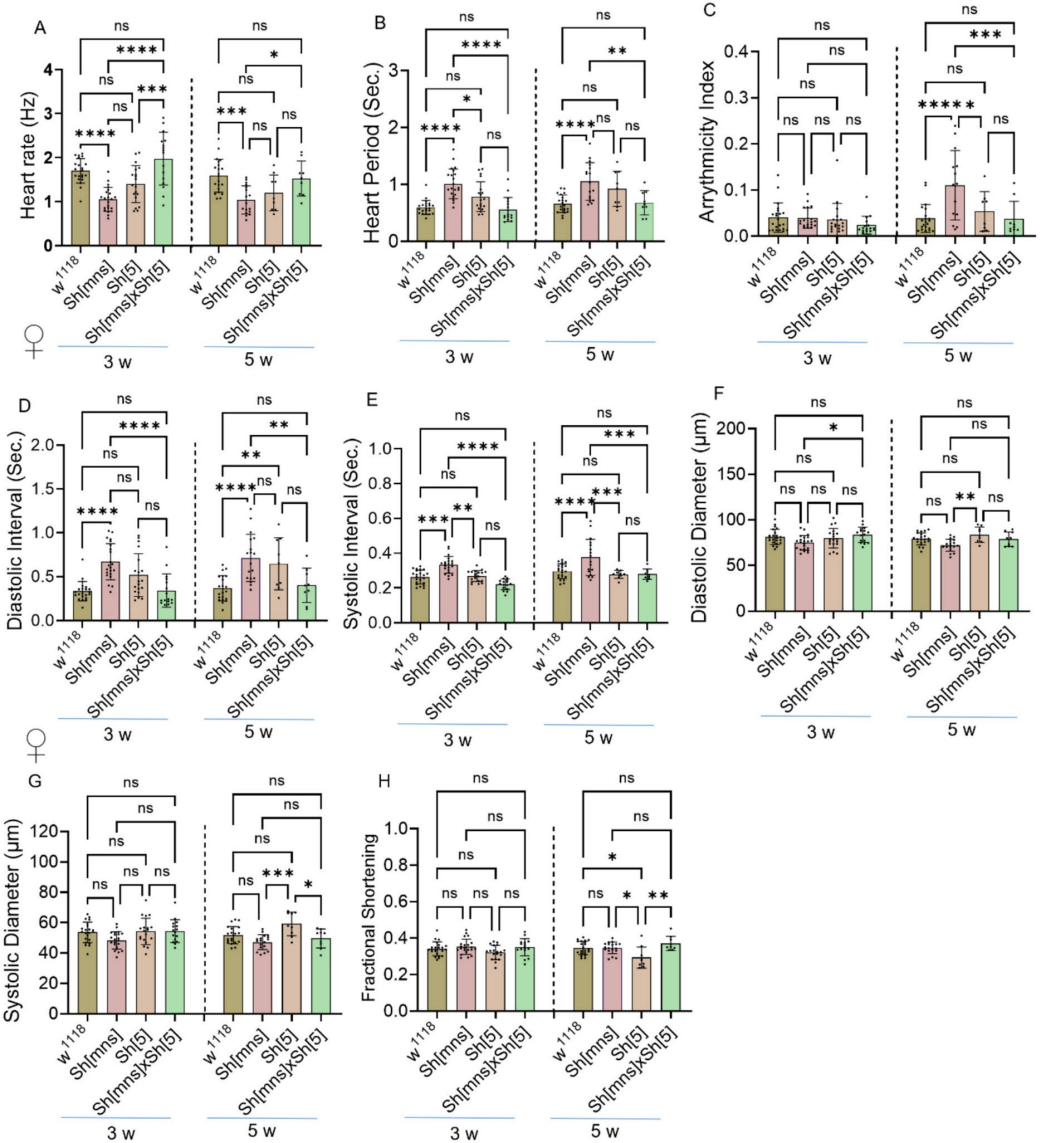
Significance of *Shaker* mutants on female heart function. **A**–**H** Represents heart physiology analysis data collected from three and five-week-old females (**A**–**H**). The w^1118^, *Sh*^*mns*^, *Sh*^5^ and *Sh*^*mns*^x *Sh*^5^ flies. Statistics: Cardiac physiology analysis performed by Two-Way ANOVA with Šidák multiple comparison test. *p* > 0.05 (ns), *p* < 0.05 (*), *p* < 0.002 (**), *p* < 0.0002 (***), and *p* < 0.0001 (****)

**Fig. 3:**
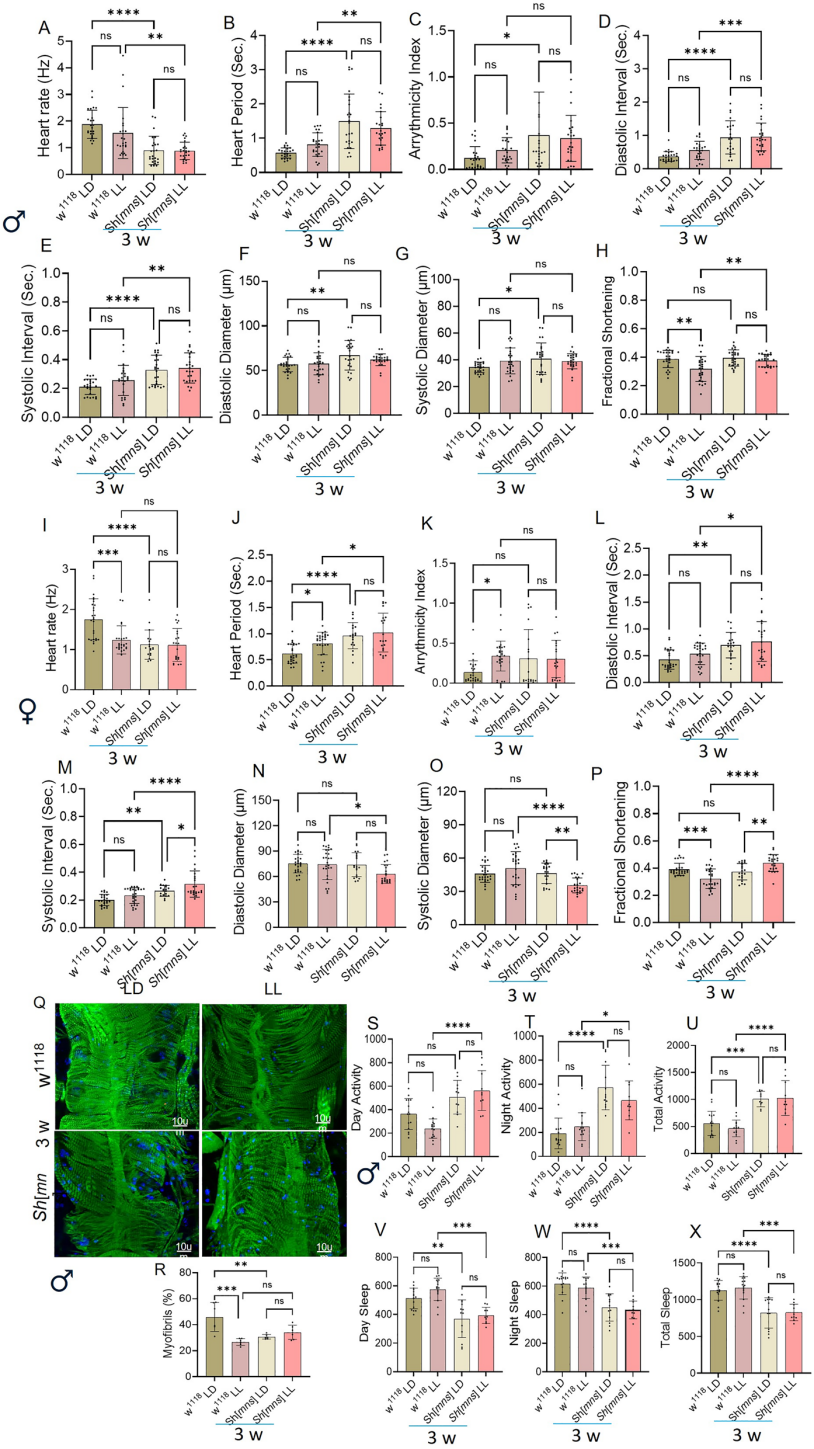
Role of circadian disruption induced by Light-Light on *Sh*^*mns*^ heart physiology and sleep/activity. Represents the impact of light/light (LL) cues-induced circadian cycle disruption on male heart physiology (**A**–**H**) and Female heart physiology under compared to age-matched light/dark (LD) (**I**–**P**). Myofibril percentage in LL w^1118^, *Sh*^*mns*^ genotypes, compared to LD (**Q**, **R**). Sleep/activity patterns of three-week-old flies (**S**–**X**). Statistics: cardiac physiology, phalloidin staining and sleep/activity analysis performed by Two-Way ANOVA with Šidák multiple comparison test. *p* < 0.05 (*), *p* < 0.002 (**), *p* < 0.0002 (***), *p* < 0.0001(****)

**Fig. 4:**
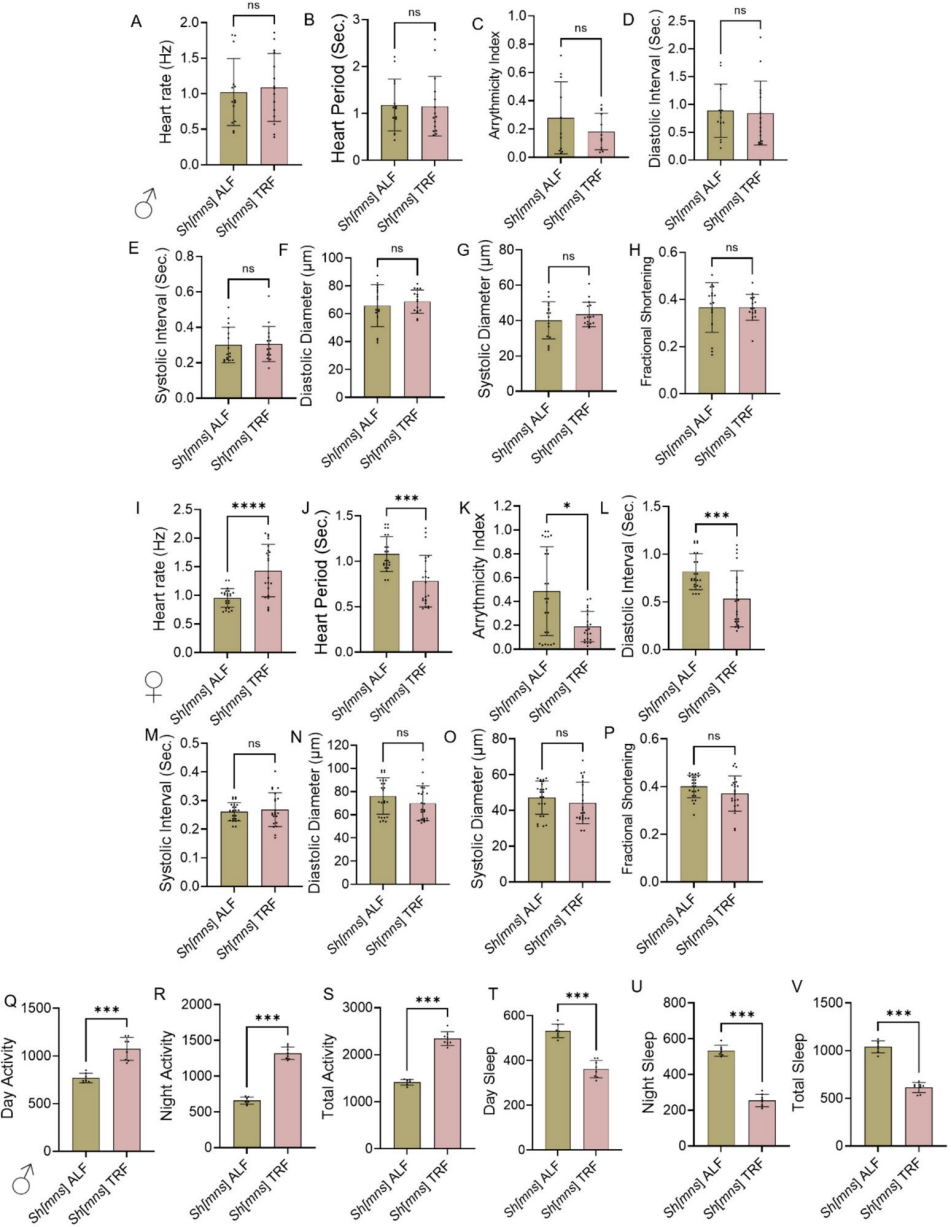
Impact of feeding times on heart physiology and sleep/activity in *Sh*^*mns*^ flies. Represents the impact of the availability of feeding times on heart function (**A**–**H**) in males and (**I**–**P**) in female flies at three-weeks of age. Sleep/activity in male flies (**Q**–**S**). Statistics: Heart physiology, and sleep/activity analysis performed by Mann–Whitney U test *p* > 0.05 (ns), *p* < 0.002 (**), *p* < 0.0002 (***)

**Fig. 5:**
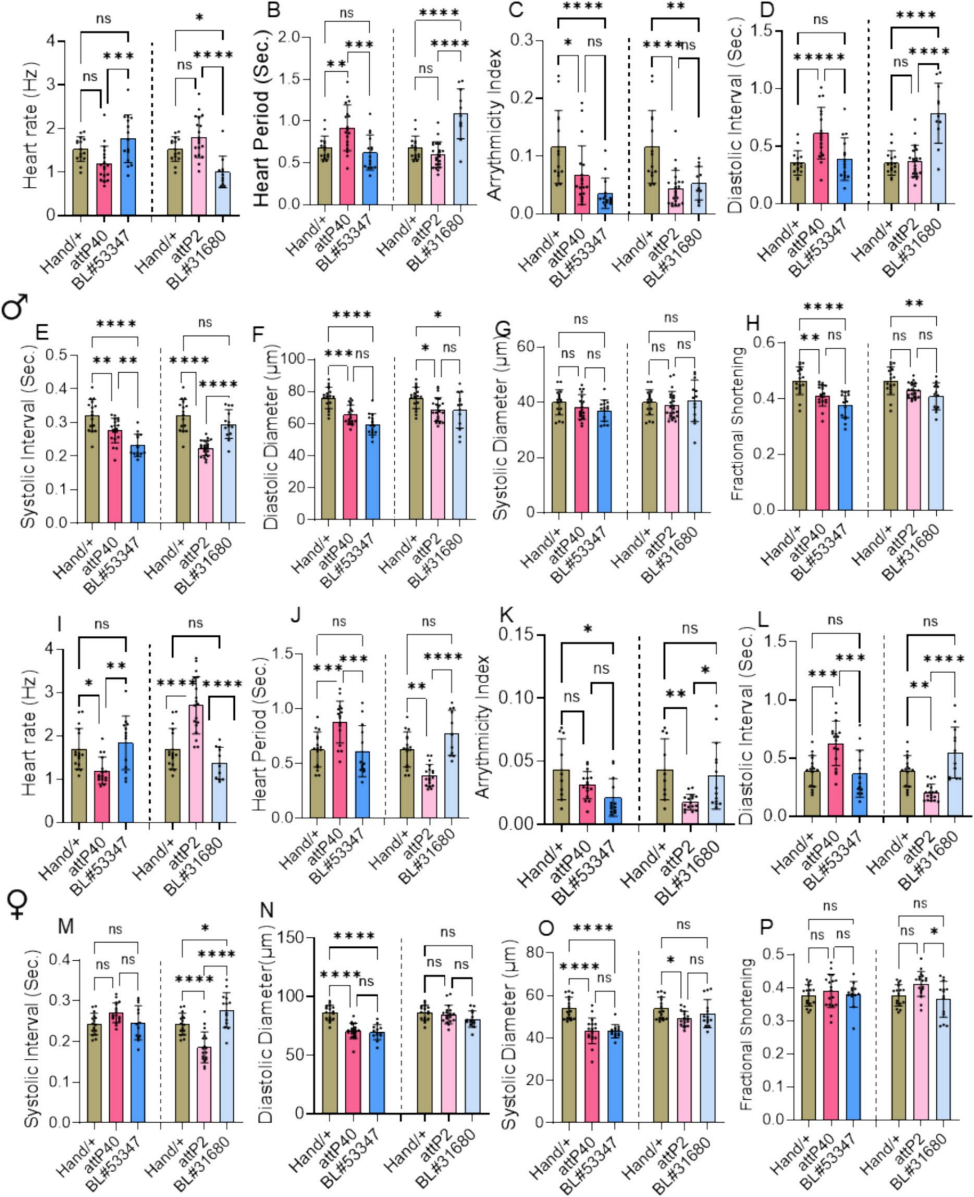
Cardiac specific expression of *Shaker* gene impacting heart function: Represents the cardiac specific knockdown of *Shaker* genes in males at three weeks (A-H) and female (I-P) three weeks flies studied here we crossed cardiac specific driver (*Hand-Gal4*) with wild type control, *Shaker* KD (BL#53347, BL#31680) genes and empty vectors for 2nd (attP40) and 3rd (attP2) chromosomes as internal controls. Statistics: Cardiac physiology analysis performed by One-Way ANOVA with Šidák multiple comparison test.. p < 0.05 (*), p < 0.002 (**), p < 0.0002 (***), p < 0.0001 (****)

**Fig. 6:**
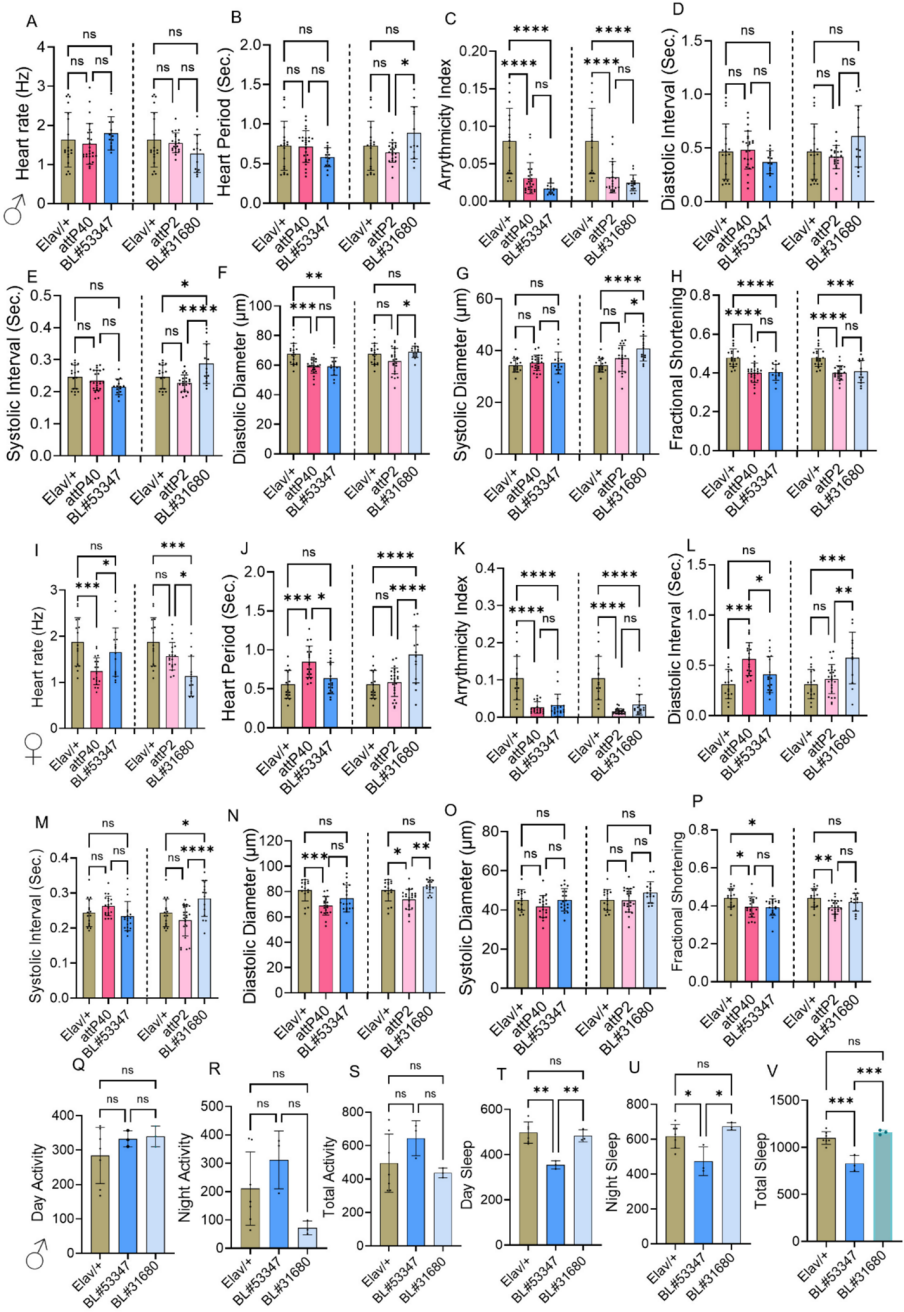
Panneuronal expression of *Shaker* gene influencing heart function. Represents the neuronal specific knockdown of *Shaker* genes in males at three weeks (**A**–**H**) and female (**I**–**P**) three weeks flies studied here we crossed panneuronal specific driver (*Elav-Gal4*) with wild type control, *Shaker* KD (BL#53347, BL#31680) genes and empty vectors for 2nd (attP40) and 3rd (attP2) chromosomes as internal controls. **Q**–**V** Sleep/activity analysis at three weeks of age. Statistics: Heart physiology analysis performed by One-Way ANOVA with Šidák multiple comparison test. *p* < 0.05 (*), *p* < 0.002 (**), *p* < 0.0002 (***), *p* < 0.0001 (****)

Statistical analysis was performed using GraphPad Prism version 10. Data from each knockdown was compared with respective controls (w^1118^, attP2, attP40, *Elav/* + or *Hand/* +) in respective experiments. For Figs. 1, 5 and 6 statistical analysis was performed with One-Way ANOVA, for Figs. 2 and 3 Two-Way ANOVA was performed to establish significance, followed by Sidak’s multiple comparisons test to assess the effects of genotype and age, light impact. For Fig. 4 Mann Whitney U test was performed to analyze the feeding timing impact on cardiac and sleep phenotypes. Data represented as mean ± SD. Statistical significance was defined as the following: *p* < 0.05 (*), *p* < 0.002 (**), *p* < 0.0002 (***), *p* < 0.0001 (****). Outlier test is performed using Z-score method (Berendrecht et al. 2023). Detailed statistical analyses among different genotypes showed in the source data.

## Results

### The Shaker *Sh*^*mns*^ mutant allele causes severe impairment of cardiac physiology, whereas the *Sh*^*5*^ mutant allele has only a subtle, age-dependent effect on cardiac performance in both male and female flies

*Shaker*^*mns*^ is a specific mutant allele of the *Shaker* gene responsible for shortened sleep duration (mini sleep mutation). These flies need significantly less sleep compared to wild-type flies (Cirelli et al. 2005). This occurs because K^+^ channels play a critical role in regulating neuronal excitability, and their dysfunction leads to hyperactivity and reduced sleep (Kim et al. 2020). The cardiac physiology and sleep/circadian activity of flies were analyzed for the *Sh*^*mns*^ and *Sh*^*5*^ (*Shaker* gene point mutation) and compared to that of the age and sex-matched *Drosophila* control line w^1118^. As shown in Fig. 1 A-H different variables of cardiac physiological data including: A. Heart rate (HR) B. Heart period (HP), C. Arrhythmicity index (AI), D, E. Diastolic (DI) and Systolic intervals (SI), F, G. Diastolic diameter (DD) and Systolic diameter (SD), and H. Fractional shortening (FS) were collected from three-week and five-week-old male flies. *Sh*^*mns*^ showed an increase in HP, AI, DI, and SI than w^1118^ and *Sh*^*5*^, as well as significantly decreased FS and HR compared to w^1118^. *Sh*^*5*^ shows a significant reduction in FS and an increase in HR compared to w^1118^*.* DD and SD did not find any significant difference between the control and mutants. The *Sh*^*mns*^ five-week flies showed an increased HP, DI, and SI than w^1118^, and *Sh*^*5*^ and AI, DD, and SD showed no change, but FS was significantly reduced compared to w^1118^ in both *Sh*^*mns*^ and *Sh*^*5*^flies. The HR was reduced in *Sh*^*mns*^ compared to w^1118^ and *Sh*^*5*^. Whereas *Sh*^*5*^ showed reduced SI, and FS compared to w^1118^. In males at three weeks (Fig. 1 I–J) phalloidin staining of the heart showed a significant difference between w^1118^ and *Sh*^*mns*^, where *Sh*^*mns*^ possesses more disorganization of actin-containing myofibrils, compared to age-matched control w^1118^. (Fig. 1 K–P), showed sleep/circadian activity analysis of w^1118^ and *Sh*^*mns*^, we observed a significant increase in activity and reduced sleep in *Sh*^*mns*^ compared to w^1118^ at three-weeks and five-weeks of age. *Sh*^*mns*^ severely impairs cardiac function and reduces sleep, with age-related worsening in males. *Sh*^*5*^ shows milder, age-dependent cardiac physiology changes. These results highlight Shaker K⁺ channels' critical role in regulating cardiac function and sleep behavior.

### Trans-heterozygous (*Sh*^*mns*^x*Sh*^*5*^) *Shaker* mutants do not aggravate cardiac physiology dysfunction linked with the *Sh*^*mns*^ mutant

We have investigated the impact of both *Sh*^*mns*^ and *Sh*^5^ alleles on female flies. (Fig. 2 A-H) represents cardiac data collected from three and five-week-old females. *Sh*^*mns*^ showed a significant increase in HP, DI, and SI compared to controls and *Sh*^*mns*^x *Sh*^*5*^ flies in both three weeks and five weeks of age. AI decreased in *Sh*^*mns*^x *Sh*^*5*^ than *Sh*^*mns*^ flies at five weeks and DD increased in *Sh*^*mns*^x *Sh*^*5*^ than *Sh*^*mns*^ flies at three weeks. *Sh*^*5*^ showed an increase in DI in five weeks compared to w^1118^. Also, *Sh*^*5*^ showed a significant increase in HP in three weeks, SI at three weeks and five weeks of age in *Sh*^*mns*^. At three weeks and five weeks of age, *Sh*^*mns*^ flies showed increased HP, DI and SI and in AI only in five weeks than w^1118^. In five weeks *Sh*^*5*^ flies AI, SI, and FS are reduced, whereas DD and SD increased than *Sh*^*mns*^. *Sh*^*mns*^x *Sh*^*5*^ flies showed decreased HP, DI, and SI at three weeks and five weeks of age and decreased AI than *Sh*^*mns*^ flies only by five weeks observed. We also found reduced SD with increased FS in *Sh*^*mns*^x *Sh*^*5*^ flies compared to *Sh*^*5*^ flies at five weeks. HR was reduced in *Sh*^*mns*^ flies at both ages, and there was no significant difference in *Sh*^*mns*^x *Sh*^*5*^ and *Sh*^*5*^ flies, but *Sh*^*mns*^x *Sh*^*5*^ flies showed a significant increase than *Sh*^*mns*^ flies at three weeks and five weeks of age. *Sh*^*mns*^x *Sh*^5^ mutants showing improved HP, DI, and SI, compared to *Sh*^*mns*^ alone at three weeks and five weeks of age, suggesting a non-additive or compensatory genetic interaction between *Shaker* alleles. Our data suggested that trans-heterozygous *Sh*^*mns*^x *Sh*^5^ mutants do not exacerbate *Sh*^*mns*^-induced cardiac defects, instead, they are partially rescuing *Sh*^*mns*^-induced heart dysfunctions.

### Light-light-induced circadian disruption further deteriorates cardiac physiological and sleep/circadian dysregulation linked with *Sh*^*mns*^ mutant

Light significantly impacts sleep-circadian cycles and cardiac function. Since the *Sh*^*mns*^ mutant flies are genetically predisposed to have shorter sleep cycles compared to w^1118^ flies, we performed cardiac physiology and sleep/circadian activity analysis to understand the impact of constant light on cardiac physiology both in male and female flies and sleep-health using male flies. (Fig. 3 A-H) represents the impact of light/dark (LD) and light/light (LL) cues-induced circadian cycle disruption on cardiac physiology on three-week-old flies. HR, HP, AI, DI, SI, DD, and SD did not show any statistically significant difference in LD vs LL condition in w^1118^ as well as in *Sh*^*mns*^ male flies. We only observed reduced FS in LL w^1118^ than LD w^1118^. During LD condition *Sh*^*mns*^ flies showed a significant increase in HP, AI, DI, SI, DD, and SD than LD w^1118^. During LL condition *Sh*^*mns*^ flies showed a significant increase in HP, DI, and SI compared to LL w^1118^, but FS was reduced.

In females (Fig. 3 I–P) at three weeks LL condition showed significantly reduced HR, FS, and increased HP, AI in w^1118^ flies compared to LD. In *Sh*^*mns*^ flies SD was reduced and FS increased significantly in LL compared to the LD condition. During LD condition *Sh*^*mns*^ flies showed increased HP, DI, SI, and reduced HR compared to LD w^1118^. During LL *Sh*^*mns*^ flies showed increased HP, DI, SI, FS, and decreased DD and SD were observed compared to LL w^1118^. The HR was reduced in LD *Sh*^*mns*^ flies and LL *Sh*^*mns*^ flies compared to respective controls in males and females, suggesting the light-light cycles regulate heart functions differently in mutant flies than w^1118^.

Phalloidin staining (Fig. 3 Q, R) indicates reduced organization of F-actin containing myofibrillar percent in heart muscles of LL w^1118^ and LL *Sh*^*mns*^ than LD w^1118^, at three weeks age, with no significant difference with LD *Sh*^*mns*^ genotype. Further we have checked the impact of heart physiology on sleep/circadian activity parameters (Fig. 3 S–X), we found day activity, night activity, and total activity were increased, and day sleep, night sleep, and total sleep were reduced in *Sh*^*mns*^ than w^1118^ flies during LD condition. During LL condition, day activity, night, and total activity increased, and sleep significantly decreased in *Sh*^*mns*^ flies than w^1118^ flies at three weeks of age. We have not observed any significant difference during LL and LD conditions between w^1118^ and *Sh*^*mns*^ flies, respectively. *Sh*^*mns*^ flies under LL conditions showed elevated HP, DI, SI and FS, reduced HR, in both male and female (Fig. 3) and sleep, (in males) with increased activity levels compared to LL w^1118^. These results indicate that disrupted light cues worsen *Sh*^*mns*^-associated physiological and behavioral impairments, highlighting the interaction between circadian regulation and Shaker channel function.

### Time-restricted feeding altered cardiac physiology and sleep/activity dysregulation linked with *Sh*^*mns*^ mutant flies

To understand the impact of feeding-fasting rhythms on heart physiology and sleep/circadian activity, on *shaker* mutant, we employed well-studied paradigm, known as time-restricted feeding (TRF) which is shown to annotate cardiac, and sleep-dysfunction linked with aging (Gill et al. 2015). Cardiac physiology under TRF and ad libitum feeding (ALF) of *Sh*^*mns*^ are shown in Fig. 4 A–H in males, and Fig. 4 I–P in females flies at three-week age. The male heart physiology did not show a significant difference between ALF *Sh*^*mns*^ and TRF *Sh*^*mns*^ male flies. Whereas in female flies, HP, AI, and DI decreased, and HR was increased in TRF *Sh*^*mns*^ flies, and SI, DD, SD, and FS did not show a significant difference compared with ALF *Sh*^*mns*^ flies. TRF partially rescues cardiac dysfunction and sleep/circadian activity disruptions in *Sh*^*mns*^ mutant flies. TRF improved heart function in females. Based on our observation in female flies, TRF improved cardiac parameters such as reduced HP, AI, and DI, and increased HR. In males, TRF did not show major changes in cardiac physiology. The sleep/circadian activity analysis (Fig. 4 Q–V) in male flies at three weeks of age are showed increased day activity, night activity and total activity (Fig. 4 Q–S), whereas day sleep, night sleep and total sleep was reduced significantly in TRF than in ALF *Sh*^*mns*^ flies (Fig. 4 T–V). These findings suggest that feeding timing can partially rescue *Shaker*-linked cardiac and sleep-circadian behavioral impairments through circadian-aligned interventions.

### Cardiac-specific knockdown of the *Shaker* gene led to compromised cardiac function

To understand cardiac-specific role of *shaker* gene, cardiac-specific knock-down of *Shaker* gene was carried out using UAS-RNAi stock *Shaker*, using Gal4-*UAS* expression system with (*Hand-Gal4*) driver. As indicated in the method section, the experimental and control RNAi fly lines were crossed with *Hand-Gal4*, and progeny were collected and aged. Heart physiology analysis performed at three-week age in male and female flies. In males (Fig. 5 A–H) BL#53347 showed significant decrease in AI, SI, DD, and FS compared to the *Hand/* + control. Second line BL#31680 showed a significant increase in HP, DI compared to *Hand/* + , and observed significant decrease in HR, AI, FS, and DD compared to *Hand/* + control. Female flies at three-weeks (Fig. 5 I–P), BL#53347 showed a significant decrease in AI, DD, and SD, compared to *Hand/* + controls. Whereas BL#31680 showed a significant increase in SI, compared to the *Hand/* + control. Cardiac-specific knockdown of the *Shaker* gene using *Hand-Gal4* disrupts heart function, with BL#53347 and BL#31680 showing altered intervals, diameters, and reduced FS. These results confirm a direct, cardiac-intrinsic role for *Shaker* in maintaining normal heart physiology. Based on observation of both BL#53347 and BL#31680 flies showing altered intervals, diameters, and reduced fractional shortening. The difference in hart physiology in two RNAi lines is due to difference in the knock-down level (60% reduction and 40% reduction at RNAi level for the BL#53347 and BL#31680 respectively). These results demonstrate that *Shaker* function in cardiomyocytes is essential for maintaining proper cardiac rhythm, contraction strength, and structural integrity in *Drosophila* cardiac physiology.

### Panneuronal knockdown of the *Shaker* gene led to altered sleep and cardiac physiology

We have revealed the significance of the *Shaker* gene in a non-cell autonomous manner in the heart, upon its knockdown in neuronal cells using the *Elav-Gal4* driver (BL#458). The experimental and control fly lines were crossed with *Elav-Gal4* driver and progeny were collected and aged for collection of cardiac physiological parameters. cardiac physiology analysis performed at three-week age in male and female flies. In males (Fig. 6 A–H) BL#53347 flies showed a significant decrease in AI, DD, and FS compared to *Elav/* + controls. Another line BL#31680 flies showed a significant decrease in SI, AI, FS parameters and significant increase in SD compared to *Elav/* + controls. Figure 6 I–P represents female flies at three weeks of age. BL#53347 flies showed a significant decrease in AI and FS, and BL#31680 flies showed a significant decrease in HR and AI and a significant increase in HP, DI, and SI compared to *Elav/* + controls. To further confirm if sleep-circadian alteration is associated with cardiac dysfunction, we performed sleep-circadian analyses using these flies (Fig. 6 Q–V) Sleep/circadian activity analysis at three-weeks showed no difference in daytime, nighttime and total activity, but day sleep, night sleep, and total sleep in BL#53347 were reduced compared to *Elav/* + controls. Whereas we did not observe a significant change in BL#31680 in sleep/circadian activity parameters. Both RNAi lines showed significant decrease in AI and FS in both genders. Increased SI, SD in BL#31680 and decreased DD in BL#53347 male flies. In females HP, DI and SI increased in BL#31680 observed. Additionally, *Shaker* knockdown via BL#53347 reduced sleep without affecting activity, highlighting the neural influence of *Shaker* on heart function and sleep regulation.

## Discussion

Voltage-gated potassium (Kv) channels are fundamental to maintaining electrical excitability in the brain and heart. In *Drosophila*, the *Shaker* (*Sh*) gene encodes the alpha subunit of a rapidly inactivating A-type Kv channel that is essential for shaping action potentials and neuronal firing patterns (Sewing et al. 1996). While Shaker channels have been widely investigated in sleep and synaptic transmission, their role in regulating cardiac physiology particularly in the context of aging and involvement of systemic cues has received less attention. This gap is essential to explore because the mammalian homolog Kv1.1 (KCNA1) is expressed in both the brain and the heart, and its dysfunction has been linked to neurological diseases and autonomic cardiac abnormalities (Glasscock et al. 2010; Humphries and Dart 2015). By using classical *Shaker* alleles (*Sh*^*mns*^ and *Sh*^*5*^), trans-heterozygotes, we have studied the impact of sleep-circadian disruption induced by constant light, LL and feeding-fasting rhythms mediated by TRF regimens. We further explored the cell-autonomous and non-cell autonomous impact of *Shaker* gene to unravel neurocardiac axis regulating sleep and cardiac function. This study confirms that *Shaker* is not only a neuronal regulator of sleep/circadian activity but also a crucial molecular link between cardiac output, and circadian and metabolic behavior.

*Sh*^*mns*^ multination previously known for their extremely short-sleeping phenotype (Wu et al. 2008). Our innovative finding demonstrated that *Sh*^*mns*^ leads to age-dependent decline in cardiac function that is strongly corelating with sleep disturbances. Specifically, in three-week-old male *Sh*^*mns*^ flies we observed significantly prolonged heart period (HP), increased arrhythmicity index (AI), extended systolic interval (SI), diastolic interval (DI) along with reduced heart rate (HR) and fractional shortening (FS). Whereas diastolic diameter (DD) and systolic diameter (SD) are largely unchanged. At five weeks male flies showed increased HP, SI, DI and worsening of HR and decreased FS (Fig. 1 A–H) confirming age associated decline in cardiac function. In contrast female *Sh*^*mns*^ at three-weeks showed similar increase in HP, SI, AI, and DI. At five-weeks females continued to show DI prolongation and significant FS reduction (Fig. 2 A–H). This reflects the progressive systolic impairments in both sexes (Ma et al. 2020). These deficits were more severe in older flies, indicating a progressive loss in cardiac integrity as reported in aging-associated electrical remodeling described in mammalian hearts with Kv channel impairments (Nattel et al. 2007). The F-actin containing myofibrillar disorganization in male flies at three-weeks age in *Sh*^*mns*^ suggesting the earlier onset of cardiac tissue structural vulnerability in males (Fig. 1 I, J). This is further increased in three-week males during LL-condition (Fig. 3 Q, R). This indicates light-driven circadian disruption may also interfere with cytoskeletal maintenance, likely through pathways that regulate muscle protein turnover and mitochondrial function (Illescas et al. 2021).

Interestingly, the *Sh*^5^ allele, which affects the same gene, causes milder and age-dependent cardiac abnormalities in both male and female flies at three- and five-weeks age (Figs. 1–2) in DI mildly and FS significantly. The sleep/circadian activity in in males at three and five weeks showed significant increase in day, night and total activity and significant decrease day, night and total sleep (Fig. 1 K–P) suggesting impact of *Sh*^*mns*^ gene mutation, on circadian functions in addition to affecting cardiac function. The genetic background interactions provide more mechanistic insights. The trans-heterozygotes (*Sh*^*mns*^/*Sh*^5^) flies do not show worsen *Sh*^*mns*^- induced cardiac defects, but they exhibit partial rescue both in three- and five-weeks females. They showed reduced HP, DI, SI compared to *Sh*^*mns*^ homozygote. These findings point to a non-additive or compensatory channel interaction. These findings align with the composition and stoichiometry of Kv channel subunits which influence channel gating properties, conductance, and phenotypic severity across neuronal and non-neuronal tissues (Pongs and Schwarz 2010). The trans-heterozygous *Sh*^*mns*^/*Sh*^5^ flies demonstrate partial recovery of *Sh*^*mns*^-induced deficits. We believe such type of allele-specific changes provide valuable genetic tools for exploring channel function under physiological and pathological conditions.

LL-induced circadian disruption light and feeding timing, mediated by TRF, acting as prominent modulators. During LL- three weeks females showed significant increased HP, DI, SI, FS and decreased DD, SD compared to LL control. This shows that circadian disruption worsens underlying channelopathies, most likely due to mis regulation of circadian genes such *Bmal*1 and *Rev-Erb*α affect Kv ion channel expression and electrophysiological functions and cardiac tissue contraction pathways (Takeda and Maemura 2015). Furthermore, circadian misalignment in cardiac tissue has been shown to enhance sensitivity to arrhythmias, remarkably when repolarization reserves are low (Hayter et al. 2021). In males HR, FS decreased and HP, DI, SI increased significantly at three weeks age. This suggesting that *Shaker* mutation showing limited or negligible variation in sex-dependent manner during stress condition. The feeding timing variation using TRF showed sex-dependent rescue in three weeks females, the TRF restored HR, shortened HP, significantly lowered AI and DI compared to ALF. In males AI is found to have a potential trend towards rescue but not confirmed in our study. TRF improves cardiac mitochondrial efficiency, lowers oxidative stress, and slows cardiac aging in both *Drosophila* and rats (Milan et al. 2024). As TRF benefits were sex-specific in our study, which suggest a need to explore how nutrient sensing and hormonal signaling intersect with Kv channel regulation in a sex-dependent manner. Further during sleep/activity analysis in males we found a severe reduction in sleep and increased day, night and total activity rhythms suggesting minimal cardiac rescue in males. This combined disturbance of sleep and cardiac function in *Sh*^*mns*^ mutants reflects what is seen in Kv1.1 knockout mice, which have seizures, impaired sleep, and autonomic cardiac dysregulation (Hu et al. 2025), supporting an evolutionarily conserved neurocardiac role of Kv channels. The TRF data supporting that TRF improves cardiac function when repolarization defects are moderate rather than extreme. It also supporting the possibility of sex dependent potassium channel conductance and interaction with genetic and environmental factors distinctly.

Tissue specific knockdown of *Shaker* gene was performed to confirm whether the observed cardiac performances were cell-autonomous or non-cell autonomous. Two independent RNAi of *Shaker* were used for the cardiac-specific knockdown using *Hand-Gal4* with BL#53347 and BL#31680 commonly affecting both male and female AI, and DD with BL#31680 SI observed commonly, but males showed significant increase in HP, DI, and decrease in HR, AI, DD, and FS compared to controls at three-weeks, confirming the direct cell-autonomous role and requirement of *Shaker* gene for cardiomyocyte contraction strength and maintaining the rhythm. Studies with mammalian system showing that Kv1.5 and Kv1.1 dysfunction leads to prolonged action potential and electrical instability (Näbauer 1998), further consolidating *Shaker* indispensable involvement in cardiac function. Neuronal knockdown of BL#53347 showed similar impact on AI, and FS in male and females, in addition males showed decreased DD compared to control at three-weeks. In BL#31680 knockdown AI and SI showed similar pattern of heart dysfunction in male and females, in addition males showed significant increase in SD and decrease in FS and females showed significant decrease in HR, increase in HP, DI, compared to control at three-weeks. These data confirm neuronal influence of *Shaker* gene on heart function when shaker conductance is reduced in brain. Our data with sleep/activity analysis showing reduced sleep in BL#53347 neuronal knockdown further supporting a possible bidirectional neuro-cardiac axis impacting the sleep quality and heart function via neural synaptic output. Earlier studies in mammals also supporting the non-cell autonomous role of Shaker ion channel (Trosclair et al. 2020). These findings emphasize the dual role of central and peripheral Shaker activity in cardiac homeostasis and demonstrate how ion channelopathies can cause multisystemic disorders due to common molecular components.

**Fig. 7:**
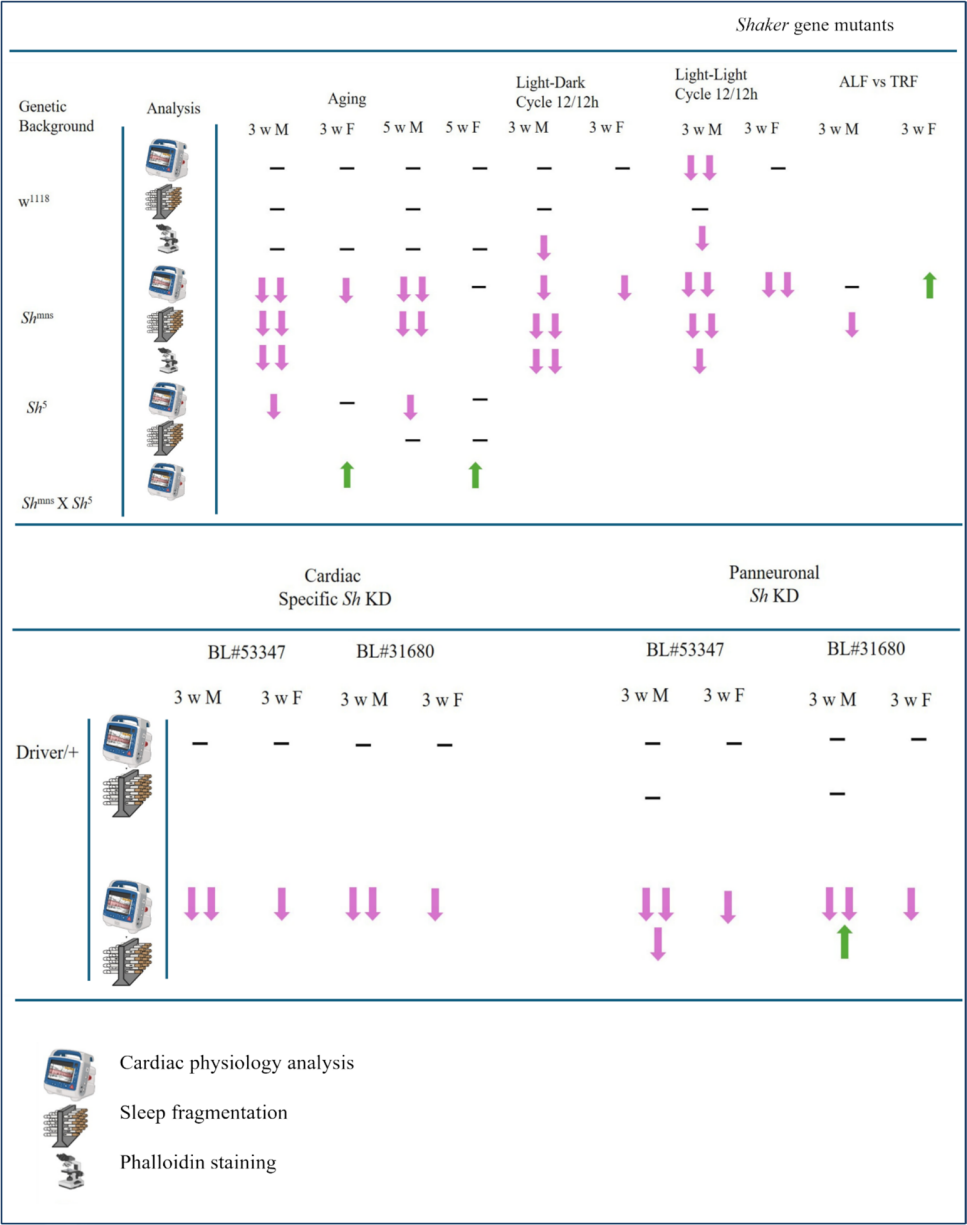
Modulation of neurocardiac function and sleep behavior in *Drosophila Shaker* mutant flies. Summary figure represents significance of *Sh*^*mns*^ and *Sh*^5^ mutants, *Sh*^*mns*^ X *Sh*^5^ Trans heterozygotes and *Shaker* gene knockdown on cardiac physiology, sleep parameters and cardiac muscle myofibril percentage (Phalloidin staining). Dash (–) represents no change, (no sign) not studied, (single up arrow) significant improvement, (single down arrow) significant decrease, (double arrow) very significant difference in actual physiological function

In our study we found that *Shaker* channel is a core integrator of sleep, circadian behavior, and cardiac physiology in *Drosophila* (Fig. 7). *Shaker* function links both nervous system and cardiac systems and their sensitivity to environmental cues, including light cycles and feeding patterns. *Shaker* mammalian homologs and its complex role places *Drosophila* in a strong position as a model system to study neurocardiac channelopathies, such as those caused by KCNA1 mutations, long QT syndromes, and sudden unexplained death in epilepsy (SUDEP). Additionally, our results highlight the significance of circadian-aligned interventions, such as TRF, to alleviate channelopathy-induced cardiac dysfunction, suggesting a non-pharmacological approach to restore physiological stability in genetically predisposed systems (Fig. 7). Potential research should investigate whether clock genes directly regulate *Shaker* transcription or post-translational modification, and how metabolic signals like AMPK or TOR act together with *Shaker* function across tissues. Understanding these regulatory connections will afford critical insights into how timing, excitability, and metabolism converge at the level of ion channels to shape organismal health.

### Limitations

*Drosophila* is a well-established model for studying the human sleep–circadian cycle and age or disease-related changes in cardiac physiology. However, *Drosophila* cardiac function is influenced primarily by glutamatergic and neuropeptidergic signals, whereas the mammalian heart relies on autonomic regulation, a specialized conduction system, and multiple chambers. Likewise, although the A-type Shaker channel in flies regulates repolarization, its role is not directly comparable to Kv1.1 loss in mice, and there is no clear anatomical equivalent between species. Therefore, cross-species comparisons are useful for identifying conserved principles of ion-channel regulation but do not represent mechanistic equivalence. Consequently, findings from fly models should be interpreted with caution when relating them to mammalian physiology, and despite their mechanistic value, direct clinical significance cannot be drawn.

## Conclusions

This study indicates that the *Drosophila* Shaker potassium channel plays a unique and integrative role in heart physiology, sleep regulation, and circadian behavior. We show that Shaker malfunction causes age-related cardiac function decline and sleep disruption and that these outcomes are influenced by genetic background, environmental cues, and eating behavior. Both cardiac and neuronal knockdowns reveal that Shaker channels contribute to heart function through cell-autonomous and non-cell autonomous processes, indicating that they are expressed in both the heart and the nervous system. Circadian disturbance worsens *Shaker*-related impairments, but time-restricted eating improves cardiac performance in a sex-dependent manner, emphasizing the role of temporal regulation in ion channel function. These findings support *Shaker*'s role as a crucial molecular link between neurophysiology, circadian regulation, and cardiovascular health, as well as *Drosophila*'s utility as a model for studying neurocardiac channelopathies and designing behavior-based therapeutics. Future research will explore molecular pathways linking *Shaker* dysfunction to cardiometabolic and neurological diseases, aiming to identify therapeutic targets for potassium channelopathies.

## Data Availability

All the raw data are provided as source data.

## Declaration

All the data are original and have not been published anywhere.
